# Exploring the Link Between Factors in Musculoskeletal Oncology Surgery Fellowships and Industry Supplemental Income

**DOI:** 10.7759/cureus.70714

**Published:** 2024-10-02

**Authors:** Alexandra H Aitchison, Philip H Khoury, William Stoeber, Albert T Anastasio, Anthony N Baumann, Mark M Cullen, Peter Monahan, Kempland C Walley, William Eward

**Affiliations:** 1 Department of Orthopedics, Duke University School of Medicine, Durham, USA; 2 Department of Orthopedics, University of Maryland School of Medicine, Baltimore, USA; 3 Department of Orthopedic Surgery, The Perse School Cambridge, Cambridge, GBR; 4 Department of Orthopedic Surgery, Duke University Medical Center, Durham, USA; 5 Department of Rehabilitation Services, University Hospitals, Cleveland, USA; 6 Department of Orthopedics, Penn State College of Medicine, Hershey, USA; 7 Department of Orthopedic Surgery, University of Michigan, Ann Arbor, USA

**Keywords:** academics and industry, industry supplemental income, musculoskeletal oncology, musculoskeletal oncology fellowship, orthopaedic surgery

## Abstract

Introduction

In the complex landscape of healthcare economics, the financial relationship between physicians and industry remains a topic of significant interest and reflection. While the dynamics of industry relationships in certain orthopedic subspecialties have been well documented, the intricacies of industry payments within musculoskeletal (MSK) oncology programs have yet to be thoroughly explored. In this study, we aim to examine the relationship between academic and industry productivity at the fellowship level. Additionally, we seek to identify the associations between the flow of industry supplemental income to orthopedic oncologists and the specific residency and fellowship training programs they are affiliated with.

Methodology

A comprehensive retrospective analysis was conducted using data from the Centers for Medicare and Medicaid Services, Scopus, and other relevant databases in 2023. This study included 19 fellowship programs across the United States, encompassing a total of 68 faculty physicians. Various variables were examined, including H-index, lifetime supplemental income, geographical location, residency affiliation, and association with top-ranked hospitals.

Results

A statistically significant correlation was found between academic and industry productivity at the individual faculty level (p = 0.003), but not at the fellowship level (p = 0.198). The top 10% of earners accounted for approximately 70% of the total lifetime supplemental income among all fellowship faculty. Additionally, there was no statistically significant association between lifetime supplemental income or academic productivity per fellowship and geographical region, nor with the ranking of affiliated residency programs or hospitals.

Conclusions

Industry productivity among individual faculty in MSK oncology fellowship programs in the United States is significantly associated with academic productivity; however, this association was not observed at the fellowship level. Furthermore, no statistically significant relationship was found regarding the region or the ranking of the affiliated residency programs or hospitals.

## Introduction

In the ever-evolving landscape of healthcare economics, there has been growing interest among the public and governments in understanding the financial relationships between physicians and industry [1]. Many physicians seek opportunities beyond their primary practice to diversify their professional experiences, support academic programs, and enhance their income. This additional income can significantly increase physician salaries and provide exposure to innovative work, enriching their professional portfolios [1]. However, it also raises concerns about potential conflicts of interest, as the line between patient advocacy and industry influence can sometimes be unclear [2,3]. To foster transparency, the Physician Payments Sunshine Act was enacted in 2010, mandating the reporting of industry-sponsored payments to physicians, which has been extensively analyzed [4,5].

Among physician specialties, orthopedic surgery stands out for its significant role in the landscape of industry-sponsored payments. A 2016 study by Iyer et al. reveals that while orthopedic surgeons comprise only 3.4% of all payment recipients, they account for an astonishing 25.6% of the total value of industry disbursements. A majority of 69% of all practicing orthopedic surgeons received these payments [6]. However, the median compensation from these payments ranges only between $100 and $300 [6]. Notably, 10% of orthopedic surgeons receive 95% of the funding, and 20% of all supplemental income has gone to four individual surgeons, totaling over $180 million [6]. Additionally, these figures can fluctuate significantly when analyzed by orthopedic subspecialties. While several reports have investigated payments received by various fellowship-trained subspecialists, the field of orthopedic oncology remains notably absent from these analyses [7-10]. Interestingly, patients tend to hold a more favorable view of surgeons with active industry relationships for research funding [6]. In contrast, surgeons who have financial ownership in a company or receive direct monetary payments, food, or travel expenses from the industry are often viewed negatively [6,7].

Musculoskeletal (MSK) oncology is one of the smaller subspecialties within orthopedic surgery. As of 2019, fellowship-trained MSK oncologists constituted only 1.5% of specialty-trained orthopedic surgeons, with just 14 accredited fellowship programs in the United States [11]. Each fellowship program varies significantly in its academic rigor, offering diverse experiences that can influence a physician's future career trajectory [12,13]. Orthopedic oncologists require a comprehensive skill set to address the complex and unique surgical needs of their patients [14,15]. They must possess a thorough understanding of the anatomy and specialized techniques related to tumors, as well as knowledge of the unique surgical devices, implants, imaging modalities, and therapeutics available to them [16,17]. This relationship underscores the need for close ties between MSK oncology and the industry, presenting opportunities for advancements in patient care.

In the present study, we aim to determine the relationship between academic and industry productivity at the fellowship level, as well as the associations between the flow of industry supplemental income to orthopedic oncologists and the specific residency and fellowship training programs they are affiliated with. This study seeks to elucidate the relationship between training-associated factors and industry payments in the field of MSK oncology. We intend to highlight the factors influencing these relationships and their broader implications for MSK oncology.

## Materials and methods

Study sources

As of April 2023, the most current industry, academic, and other relevant variables associated with MSK oncology orthopedic surgery fellowships in the United States were collected. For data regarding faculty physician lifetime supplemental income (in dollars and payments), the present study utilized the Centers for Medicare and Medicaid Services (CMS) website. Individual faculty physician information from the CMS was aggregated to represent data from each fellowship program. The H-index was employed as a measure of academic productivity, generated through Scopus for both individual and combined fellowships [18].

Information on MSK oncology orthopedic surgery fellowship programs was sourced from the American Association of Hip and Knee Surgeons (AAHKS) website. Data was gathered on each individual fellowship from their respective websites, as per the information available on the AAHKS platform. Additionally, Newsweek was consulted for affiliated hospital rankings, while Doximity, filtered by reputation, was used for rankings of affiliated orthopedic surgery residency programs.

Data extraction

Data extraction was conducted by a single author. The collected data encompassed the following variables: total combined lifetime supplemental income per fellowship (in dollars), total H-index per fellowship, combined total supplemental income payments per fellowship, average total lifetime supplemental income per faculty physician per fellowship (in dollars), type of program (university, mixed, or private), Doximity ranking of affiliated orthopedic residency programs (top 10, top 11-30, top 31, and unranked), geographic region of the fellowship program (Northeast, Midwest, Southeast, Southwest, and West Coast), and Newsweek ranking of affiliated hospitals (top 10, top 11-30, top 31, and unranked).

Study definitions

The Doximity ranking of affiliated orthopedic residency programs was linked to the orthopedic oncology fellowships through direct affiliation via shared names (e.g., Mayo Clinic Orthopedic Surgery Residency and Mayo Clinic Minnesota Musculoskeletal Oncology Fellowship). For both Doximity and Newsweek rankings, fellowships designated as “unranked” were either associated with an unranked residency or hospital, or they were not affiliated with any at all. Total lifetime supplemental income for physicians included only reimbursements and/or money directly received by the physician categorized as “general payments” on the CMS website; research funding was excluded from the total lifetime supplemental income per fellowship. Regarding the classification of fellowship programs, “university” referred to programs directly affiliated with a university by shared name, “private” indicated programs not affiliated with any university, and “mixed” described private programs with indirect but recognized ties to a university.

Statistical analysis

The statistical analysis for this study was conducted using IBM SPSS Statistics for Windows, Version 29.0 (Released 2022; IBM Corp., Armonk, NY, USA). The Shapiro-Wilk test assessed the normality of the data, and nonparametric tests were employed due to the small sample size and characteristics of the data. Medians across three or more groups were compared using the independent-sample median test, followed by post hoc Bonferroni correction. Spearman’s rho was utilized for correlation statistics, reflecting the nonparametric nature of the data. A statistical significance threshold of p < 0.05 was established for this study. Descriptive statistics, including mean and median values, were used to represent the data.

## Results

Initial search results

As of April 2023, there were a total of 19 MSK oncology orthopedic surgery fellowship programs in the United States, comprising 72 physician faculty members. The study included only those faculty physicians for whom complete supplemental income and payment data from the CMS and H-index information from Scopus were available, resulting in a sample size of 68 physicians (94.4%). Four physicians (5.5%) lacked supplemental income and payment information on the CMS website and were excluded from the analysis. Consequently, all 19 MSK oncology orthopedic fellowship programs (100%) were included in the analysis.

Individual faculty physician demographics

The median H-index for individual physicians (n = 68) was 16.50, with a mean of 19.53 ± 15.51, and a range of 1 to 69, serving as a measure of academic productivity. In contrast, the median lifetime supplemental income for these physicians was $5,169.85, with a mean of $40,813.60 ± $89,018.15, and a range of $42.57 to $459,451.20, which reflects industry productivity. The median number of lifetime payments per individual physician was 24.00, with a mean of 43.76 ± 61.14, and a range of 1 to 333.

A statistically significant, weak, positive correlation was observed between individual physician H-index and lifetime supplemental income (p = 0.003; Spearman’s rho = 0.357). Notably, the top 10% of individual earners in terms of lifetime supplemental income (n = 7) received 69.34% of the total lifetime supplemental income ($1,924,423.16) distributed among all faculty members at MSK oncology fellowship programs (n = 68; total: $2,775,325.05).

Oncology orthopedic surgery fellowship demographics

As of April 2023, the median combined H-index of physicians per MSK oncology orthopedic surgery fellowship (n = 19 fellowships) was 55.00, with a mean of 69.89 ± 45.81 and a range of 17.00 to 217.00. The median combined lifetime supplemental income for physicians was $74,183.30, with a mean of $146,069.74 ± $177,702.63 and a range of $493.23 to $591,062.40. The median number of payments per fellowship was 110.00, with a mean of 156.63 ± 136.78 and a range of 12.00 to 490.00.

The median lifetime supplemental income per faculty member per fellowship was $16,776.40, with a mean of $40,491.33 ± $46,034.27 and a range of $246.62 to $163,567.37. The median number of faculty physicians at MSK oncology orthopedic surgery fellowships was 3.00, with a mean of 3.58 ± 1.57 and a range of 2 to 8 physicians. There was no statistically significant correlation between the combined fellowship H-index and total lifetime earnings per fellowship (p = 0.198, Spearman’s rho = 0.309). The information on the top 10 MSK oncology orthopedic surgery fellowships is presented in Table 1.

**Table 1:** Top 10 MSK oncology orthopedic surgery fellowships in the United States, ranked by combined total lifetime supplemental income, as reported on the CMS website Reported data includes total lifetime supplemental income (dollars) per fellowship, total lifetime payments per fellowship, combined H-index per fellowship, and total lifetime supplemental income per faculty physician per fellowship (dollars). CMS, Centers for Medicare and Medicaid Services; MSK, musculoskeletal

Physician ranking	Combined H-Index per fellowship	Combined lifetime supplemental income per fellowship	Combined total payments per fellowship	Supplemental income per physician per fellowship
1	217	$591,062.40	490	$73,882.80
2	73	$490,702.11	104	$163,567.37
3	53	$363,370.79	370	$90,842.70
4	55	$316,369.17	250	$105,456.39
5	53	$270,177.58	228	$90,059.19
6	105	$179,532.27	183	$44,883.07
7	17	$131,088.05	189	$65,544.03
8	61	$107,965.51	136	$35,988.50
9	145	$83,797.74	379	$11,971.11
10	43	$74,183.30	24	$37,091.65

Industry productivity by region

**Figure 1:**
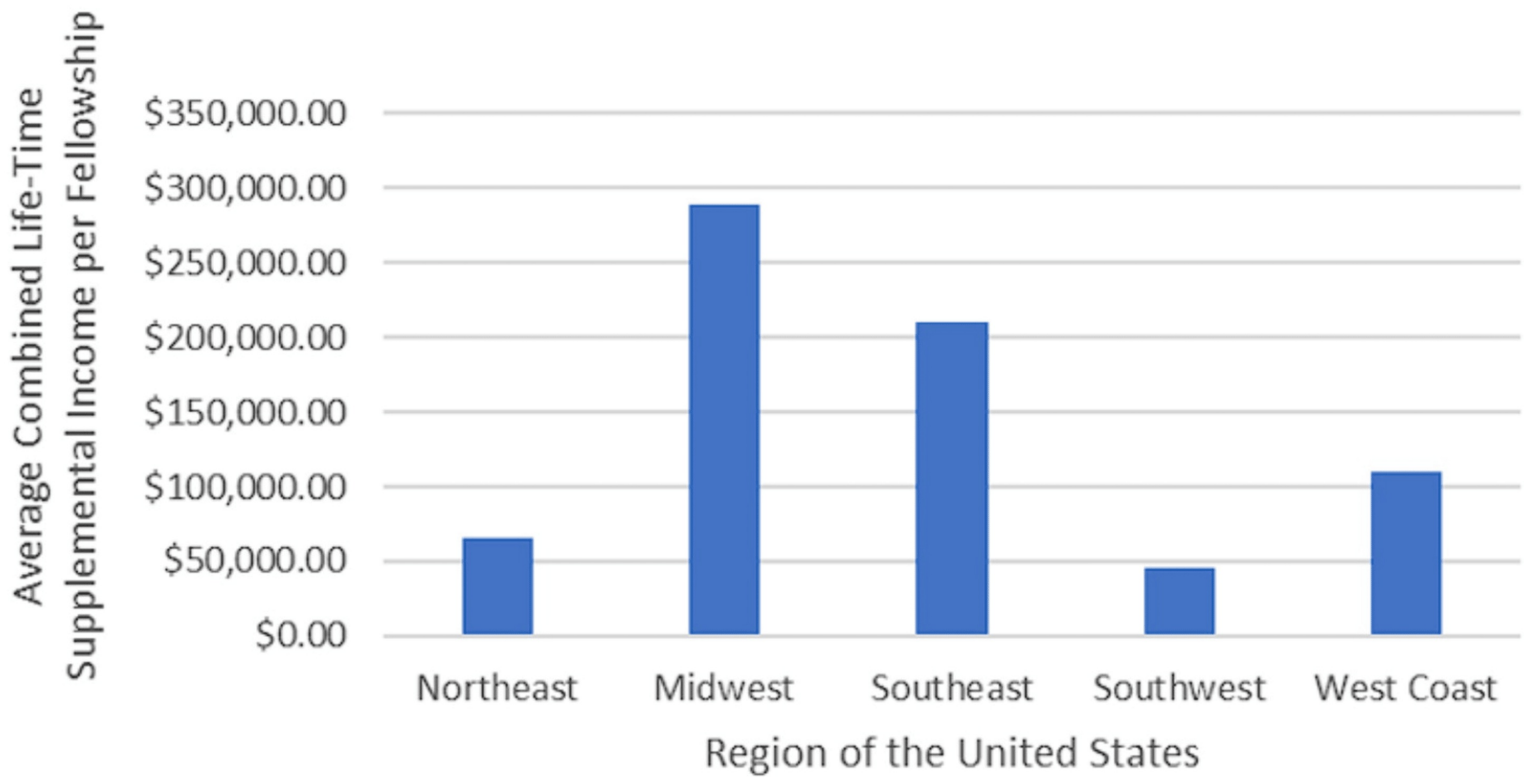
Median combined lifetime supplemental income per fellowship by region The average total lifetime supplemental income per fellowship for oncology orthopedic surgery programs in the United States is analyzed across five regions: Northeast, Midwest, Southeast, Southwest, and West Coast. The findings indicate no significant differences in total lifetime supplemental income among these regions.

Total lifetime supplemental income per fellowship showed no significant association with the region of the MSK oncology orthopedic surgery fellowship programs in the United States (p = 0.683). The median total lifetime supplemental income for fellowships in the Northeast (n = 5) was $27,531.10 (mean: $65,737.53 ± $67,104.45), while the Midwest (n = 3) reported a median of $270,177.58 (mean: $288,513.32 ± $293,810.62). In the Southeast (n = 5), the median was $131,088.05 (mean: $210,272.12 ± $207,198.00); the Southwest (n = 2) had a median of $45,455.53 (mean: $45,455.53 ± $54,224.07); and the West Coast (n = 4) reported a median of $60,981.68 (mean: $109,706.44 ± $145,831.15) (Figure 1). Additionally, no significant association was found between the average total lifetime supplemental income per faculty physician per fellowship and the region of the fellowship program (p = 0.611).

Academic productivity by region

**Figure 2:**
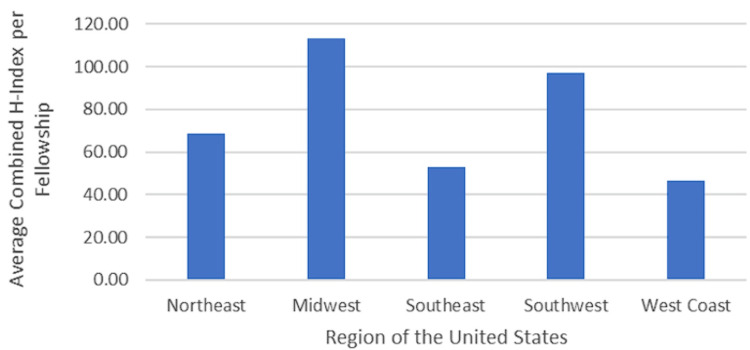
Median combined H-Index per fellowship by region The regions of the United States represented included the Northeast, Midwest, Southeast, Southwest, and West Coast.

No significant association was found between the total H-Index per fellowship and the region of the fellowship program in the United States (p = 0.793). Regarding the average total H-Index per fellowship, those in the Northeast (n = 5 fellowships) had a median H-Index of 75.00 (mean: 68.60 ± 25.89). Fellowships in the Midwest (n = 3) had a median H-Index of 70.00 (mean: 113.33 ± 90.18), while those in the Southeast (n = 5) reported a median H-Index of 53.00 (mean: 52.80 ± 26.99). Fellowships in the Southwest (n = 2) had a median H-Index of 97.00 (mean: 97.00 ± 67.88), and those on the West Coast (n = 4) had a median H-Index of 55.00 (mean: 46.75 ± 13.72) (Figure 2).

Fellowship characteristics by affiliated orthopedic residency program

**Figure 3:**
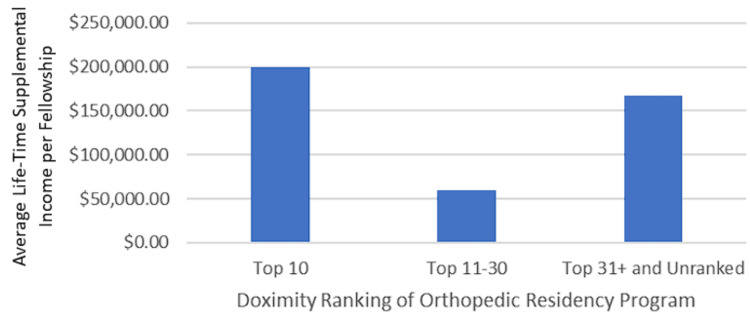
Median lifetime supplemental income per fellowship by Doximity ranking of the affiliated orthopedic residency program The categories included top 10, top 11-30, top 31+, and not ranked/not affiliated with an orthopedic residency program.

For fellowships directly affiliated with a named orthopedic residency program, no significant association was observed between total lifetime supplemental income per fellowship and the Doximity ranking of the affiliated orthopedic residency program (top 10, top 11-30, top 31+, and unranked) (p = 0.929) (Figure 3). Fellowship programs associated with a top 10 orthopedic surgery residency program on Doximity (n = 4) reported a median total lifetime supplemental income of $97,701.37, with a mean of $200,115.75 ± $271,936.98. In contrast, fellowships linked to top 11-30 orthopedic surgery residency programs (n = 5) had a median total lifetime supplemental income of $50,329.19 and a mean of $59,397.86 ± $58,702.58. Fellowships associated with programs ranked 31 or lower, or unranked/not affiliated with an orthopedic surgery residency (n = 10), reported a median total lifetime supplemental income of $78,990.52, with a mean of $167,787.28 ± $176,009.60.

Fellowship characteristics by affiliated Newsweek hospital ranking

**Figure 4:**
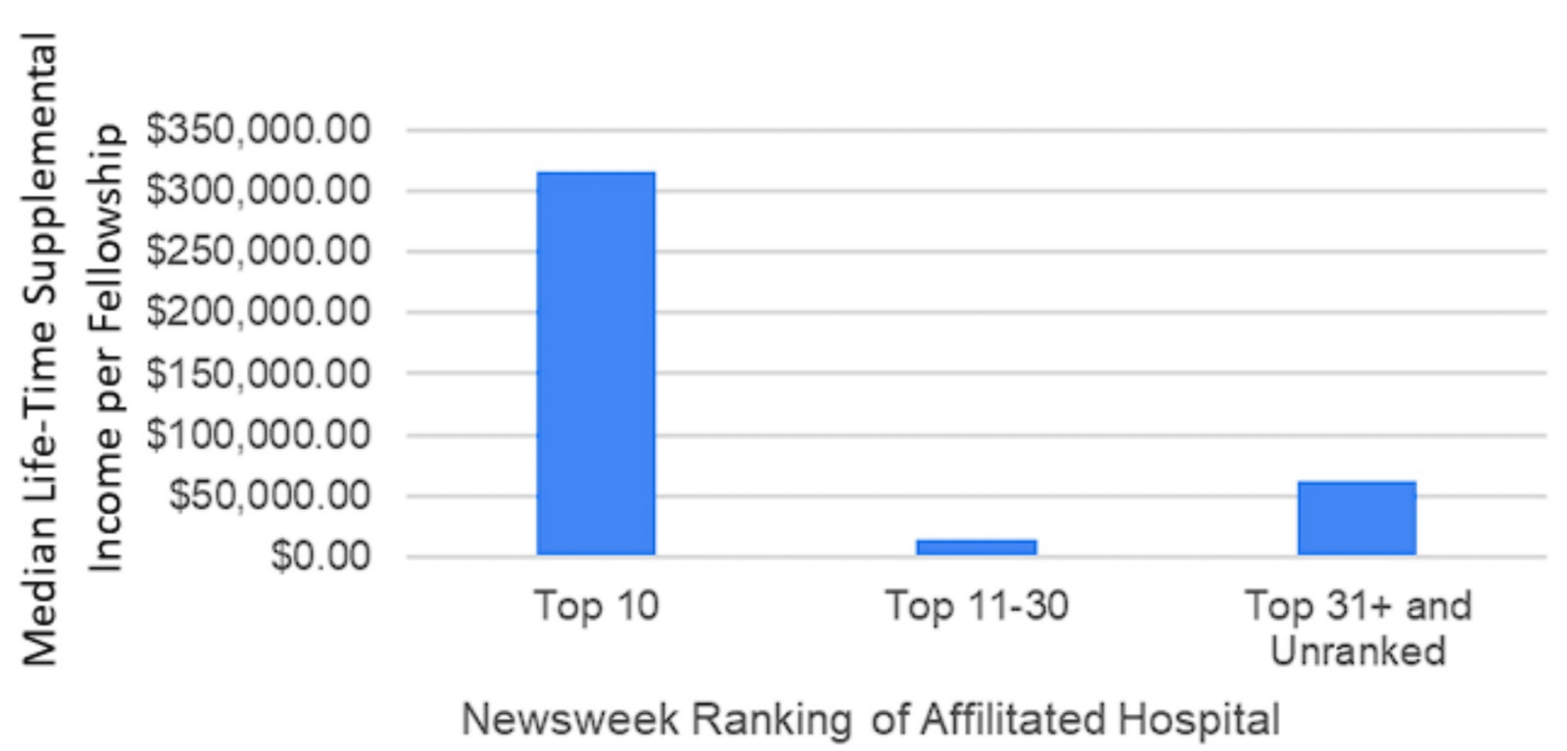
Median lifetime supplemental income per fellowship by Newsweek ranking The categories were top 10 hospitals, top 11-30 hospitals, and top 31+ and unranked/not affiliated with a hospital.

No statistically significant association was found between total lifetime supplemental income per fellowship and the rankings of directly affiliated hospitals on the Ranked Hospital Newsweek List (top 10, top 11-30, top 31+, and unranked) (p = 0.117). Fellowships associated with the top 10 hospitals (n = 3) reported a median total lifetime supplemental income of $316,369.17, with a mean of $362,321.28 ± $209,578.05. In comparison, fellowships affiliated with the top 11-30 hospitals (n = 4) had a median total lifetime supplemental income of $14,934.16 and a mean of $35,533.45 ± $48,553.57. Fellowships associated with the top 31+ hospitals or unassociated with ranked hospitals (n = 12) reported a median total lifetime supplemental income of $62,256.25, with a mean of $128,852.28 ± $159,762.67 (Figure 4).

Fellowship characteristics and type of fellowship program

No significant association was found between total lifetime supplemental income per fellowship and the type of program (university, mixed, or private) (p = 0.622). Similarly, there was no significant association between the total H-Index per fellowship and the type of program (university, mixed, or private) (p = 0.711).

## Discussion

The financial relationship between physicians and industry is a complex and multifaceted issue with both potential benefits and drawbacks [19]. The rationale for physicians receiving industry payments often centers on the mutual benefits derived from such collaborations [20]. Physicians gain access to additional resources and expertise, while industry partners can leverage physicians’ clinical and research insights to enhance their implants and devices [4]. In orthopedic oncology, this relationship is particularly intriguing due to the specialized nature of the work and the unique products and relationships involved. To our knowledge, this is the first study investigating this relationship.

Our study found a statistically significant association between academic productivity and industry productivity at the individual fellowship faculty level; however, this association disappeared at the combined fellowship level. This suggests that while financial relationships with industry may pose risks for conflicts of interest and dependence on academic progression at the individual level, the overall analysis at the fellowship level shows minimal influence from these risks. Notably, approximately 70% of industry earnings were concentrated among the top 10% of faculty within fellowship programs, indicating a potentially skewed distribution.

Interestingly, our study revealed no statistically significant relationship between geographical regions and the magnitude of industry earnings. However, physicians in the Midwest received a higher lifetime supplemental income compared to other US regions, which aligns with prior research consistently identifying the Midwest as the region with the highest reported values of industry payments across various orthopedic subspecialties, including general orthopedics, adult joint reconstruction, and foot and ankle [4,10,21]. Several factors may contribute to this discrepancy, such as the reputation of prominent institutions in the region, like the Mayo Clinic in Minnesota, the Cleveland Clinic in Ohio, the University of Chicago, and Northwestern in Illinois. These institutions may attract a higher patient volume, thereby increasing industry engagement. Although it remains unclear why the Midwest may have heightened demand for MSK oncology services, the proximity to major industry stakeholders could also be a contributing factor [10]. Nevertheless, these explanations are speculative, and further research is warranted to explore the impact of geographical factors on industry payments.

There was no significant correlation between the H-index, a measure of academic productivity, and industry payments when evaluated collectively by the institution. This finding is somewhat surprising, as we hypothesized that industry partners would be more inclined to collaborate with physicians who have a strong research portfolio and substantial scholarly impact [18]. However, this relationship might be underpowered. We also found a weak but significant association between the H-index of individual orthopedic oncologists and industry supplemental income. Additionally, no significant relationship was observed between the ranking of affiliated residency programs and industry payments, despite the established role of industry in medical training [22]. Major medical education bodies indicate a growing reliance on industry support for medical education [23], which suggests that industry support may be more prevalent where residents are present [24]. However, our data did not support this assumption. Previous studies indicated that affiliation with either a major medical school or a US top 50 ranked medical school was associated with high industry payments [25,26], suggesting that this factor may exert a more significant influence than residency program affiliation.

Another notable finding of our study was the lack of association between Newsweek hospital rankings and total lifetime supplemental income per fellowship. This contrasts with existing literature in other medical specialties, which suggests that higher-ranked hospitals often attract more industry funding [27]. This discrepancy may be attributed to the prestige associated with these institutions, the high-quality research they produce, or their potential influence on medical practice and policy. For industry partners, investing in top-tier institutions is likely seen as a strategy to ensure a higher return on investment, given these centers’ reputations for cutting-edge research and high-quality patient care [28]. The prestige of these institutions may also lend credibility to industry products and services, fostering a mutually beneficial relationship.

It is often suggested that salaries at top-ranked institutions may be comparatively lower due to the greater potential for supplemental income through industry collaborations. While this perception has not yet been thoroughly explored in the literature, our findings lend credence to this notion, indicating that physicians affiliated with higher-ranked institutions tend to receive more substantial industry payments. This could significantly influence physicians’ decisions when choosing fellowship programs and might also shape their career trajectories, as the prospect of supplemental income may offset lower base salaries at prestigious institutions.

Several limitations of this study warrant discussion. First, the cross-sectional nature of the data limits our ability to establish causal relationships between industry payments and various factors such as academic productivity, geographical location, and institutional reputation. Second, our study relied on publicly available data from the CMS Open Payments Database, which depends on accurate reporting and may not capture all forms of industry engagement or financial relationships. Third, while the H-index is a widely accepted measure of academic productivity, it has limitations, including potential self-citation and variations across different databases. Fourth, we did not account for individual physician characteristics, such as years of experience, which could also impact industry payments. Additionally, many of the statistical associations in this study may be underpowered due to the relatively small number of MSK oncology fellowship programs in the United States, necessitating caution in interpreting the results. Lastly, the study did not consider the influence of institutional policies on industry engagement, which can vary widely and may significantly affect the observed trends.

## Conclusions

Academic productivity may exhibit a statistically significant association with industry productivity at the individual level among faculty physicians in MSK oncology fellowship programs in the United States; however, this association was not evident at the fellowship level. Notably, the top 10% of earners accounted for approximately 70% of the supplemental income, indicating a skewed distribution. Furthermore, no statistically significant association was found between academic or industry productivity and geographical region or the rankings of affiliated hospitals or orthopedic surgery residency programs. By understanding these dynamics, we can gain deeper insights into the complexities of industry-physician relationships, particularly in specialized fields like MSK oncology, where the stakes for innovation and quality care are particularly high.
